# One-Step Continuous Fabrication of Solid, Porous and Hollow YAG:Eu^3+^ Microspheres by Ultrasonic Spray Pyrolysis: A Rare-Earth-Saving Strategy for Red-Emitting Phosphors

**DOI:** 10.3390/ma19214502

**Published:** 2026-10-26

**Authors:** Yifu Liu, Shuang Deng, Qiuying Wang, Yuan Wang, Jinnan Wang

**Affiliations:** 1 School of Civil Engineering and Architecture, Keyi College of Zhejiang Sci-Tech University, Shaoxing 312369, China; lyf1571955524@ky.zstu.edu.cn (Y.L.); qiuying.w.wang@ky.zstu.edu.cn (Q.W.); 2 School of Architectural Engineering, Kaili University, Kaili 556011, China

**Keywords:** rare-earth saving, YAG:Eu^3+^, hollow microspheres, ultrasonic spray pyrolysis, morphology control, red phosphor

## Abstract

Reducing the consumption of scarce and costly rare-earth elements without sacrificing emission performance is a recognised challenge for the sustainable production of phosphors. Hollowing the phosphor particle is an attractive route to this goal, because in micro- and sub-micron phosphors the rare-earth ions that contribute most to the emission reside mainly in the surface shell. However, existing hollow-phosphor routes (template, hydrothermal and sol–gel) are multi-step, batch-type and difficult to operate continuously, and they have rarely been applied to yttrium aluminium garnet (Y_3_Al_5_O_12_, YAG), a technologically important but hard-to-crystallise host that conventionally requires solid-state firing above 1600 °C. Here we report a one-step, continuous ultrasonic spray pyrolysis (USP) route that fabricates solid, porous and hollow YAG:Eu^3+^ microspheres from the same general process at only 900 °C, using citric acid as a pore-forming agent and sucrose as a film-forming agent. Single-phase cubic YAG was obtained for all three morphologies without any post-annealing, and the surface porosity and shell thickness varied with the additive concentration. On the same particle population, the hollow microspheres (H3) retained 96.1% of the relative emission intensity of their solid counterparts under 250 nm excitation while consuming only ~57% of the rare-earth-bearing raw material—a ~43% saving geometrically estimated with a spherical-shell volume model at the measured mean outer diameter and shell thickness—corresponding to a geometrically estimated ~1.68-fold gain in emission per unit of rare-earth input. Absolute quantum-yield measurements indicate that the hollow shell preserves the luminescence efficiency of the solid particle (27.97% versus 29.37% under 250 nm excitation), so the retention of almost the full emission with only ~57% of the material is consistent with the removed core being largely optically inactive rather than any efficiency penalty. A shell-volume model further projects that the saving would increase to ~77–80% for larger thin-walled spheres, although the hollow powders obtained here have mean diameters below 1 μm. The route is simple and compatible with continuous processing, and offers a potentially general, material-saving strategy for the production of rare-earth phosphors and other functional oxides.

## 1. Introduction

Rare-earth-activated phosphors underpin solid-state lighting, displays and a wide range of optoelectronic devices, yet they depend on rare-earth elements whose supply is concentrated, costly and increasingly regarded as critical [1,2]. As demand grows, lowering the rare-earth content of phosphors—without degrading their optical performance—has become an explicit and widely acknowledged target for sustainable luminescent-materials research [2,3]. Among red-emitting hosts, Eu^3+^-doped yttrium aluminium garnet (Y_3_Al_5_O_12_, YAG) is attractive because it combines the chemical and thermal robustness of the garnet lattice with the efficient orange–red ^5^D_0_–^7^F_J_ emission of Eu^3+^, and is of interest for field-emission displays, mercury-free fluorescent lamps and other ultraviolet-excited luminescent devices [4,5,6].

For micro- and sub-micron phosphors, which still dominate practical use because of the high surface-defect density and low efficiency of nanophosphors [7], the rare-earth ions that effectively contribute to the emission are concentrated in the surface layer of each particle. This observation suggests a direct material-saving opportunity: if the optically inactive core of a solid particle is removed, a hollow particle of the same outer size should retain most of the emission while consuming far less rare-earth raw material [8]. Replacing solid phosphor particles with hollow or porous analogues is therefore attractive from the standpoint of raw-material efficiency.

Hollow and porous rare-earth luminescent microspheres have indeed been prepared, mostly by template [9], hydrothermal [10] and sol–gel [11] methods. These routes, however, share three practical drawbacks: they are multi-step (often requiring template synthesis, coating, ion exchange and template removal), they operate in batch mode with long cycles, and they consume large volumes of solvent, all of which hinder continuous, scalable production [12]. Spray pyrolysis, by contrast, converts atomised precursor droplets directly into spherical particles in a single continuous pass, offering high purity, uniform size and excellent stoichiometric control [13,14]; the droplet-to-particle conversion can be steered towards solid, porous or hollow morphologies by tailoring the precursor chemistry [15]. Cho et al., for example, obtained yolk–shell Y_2_O_3_:Eu^3+^ powders by spray pyrolysis with sucrose and showed that the resulting void enhances light scattering and emission [16]. Nevertheless, two gaps remain. First, such studies target the easily crystallised Y_2_O_3_ host and aim primarily at brightness enhancement, rather than at quantifying rare-earth saving. Second, the approach has not been demonstrated for YAG, a host that is notoriously difficult to crystallise: conventional solid-state synthesis of phase-pure YAG requires firing above 1600 °C and passes through intermediate YAP and YAM phases [4,15], which makes the low-temperature, single-step formation of morphology-controlled YAG microspheres a genuine challenge.

Here we address these gaps with a one-step, continuous USP route that fabricates solid, porous and hollow YAG:Eu^3+^ microspheres from the same general process at only 900 °C. Two inexpensive organic additives serve complementary roles: citric acid is thought to chelate the metal ions and, on thermal decomposition, leaves a uniform mesoporous texture (pore-forming agent), whereas sucrose is thought to form a surface film during droplet drying that drives the solute outward and creates a central cavity (film-forming agent); the additive-free precursor yields solid spheres. This work makes four contributions. (i) It realises phase-pure cubic YAG:Eu^3+^ microspheres at 900 °C—far below the conventional >1600 °C—without any post-annealing. (ii) It achieves solid, porous and hollow morphologies in a single, unified process, with the porosity and shell thickness varying with additive concentration. (iii) It targets rare-earth saving explicitly and quantifies it with a spherical-shell volume model: evaluated on the same particle population used for the optical measurements, the hollow H3 architecture retains 96.1% of the emission while consuming only ~57% of the rare-earth-bearing raw material (a ~43% saving), yielding a geometrically estimated ~1.68-fold gain in emission per unit of rare-earth material, with the hollow shell preserving the absolute quantum yield of the solid particle (27.97% versus 29.37%), with the shell-volume model projecting the saving rising to ~77–80% for larger thin-walled spheres. (iv) The process is compatible with continuous operation and potentially scalable, in contrast to multi-step template and hydrothermal routes. Beyond YAG:Eu^3+^, the strategy should be transferable to other rare-earth phosphors and functional oxides.

## 2. Experimental Section

### 2.1. Materials

Yttrium nitrate (Y(NO_3_)_3_·6H_2_O, AR), aluminium nitrate (Al(NO_3_)_3_·9H_2_O, AR), europium nitrate (Eu(NO_3_)_3_·6H_2_O, AR), sucrose (C_12_H_22_O_11_, AR) and anhydrous citric acid (C_6_H_8_O_7_, AR) were used as received. Deionised water was used throughout.

### 2.2. Synthesis of YAG:Eu^3+^ Microspheres

All samples were prepared in an ultrasonic spray pyrolysis apparatus operating continuously, with a total precursor concentration fixed at 10 wt%. Three groups of products were designed (Table 1): additive-free solid microspheres (group S), porous microspheres prepared with citric acid (group P), and hollow microspheres prepared with sucrose (group H). To optimise the activator content, additive-free precursors with Eu^3+^ molar fractions of 1, 3, 5 and 7 mol% (S1, S3, S5, S7) were prepared. For the porous and hollow series the Eu^3+^ content was fixed at the optimum 5 mol% and the citric acid (P1, P3, P5) or sucrose (H1, H3, H5) concentration was set to approximately 5.6, 16.7 and 27.8 g L^−1^, respectively.

Taking S1 as an example, 7.52 g Y(NO_3_)_3_·6H_2_O, 12.40 g Al(NO_3_)_3_·9H_2_O and 0.07 g Eu(NO_3_)_3_·6H_2_O were dissolved in 180 mL deionised water under stirring for 30 min. The solution was atomised by an ultrasonic nebuliser (1.7 MHz) into fine droplets, which were carried by a gas flow into a tube furnace held at 900 °C. There the solvent rapidly evaporated, the precursors decomposed, and single-phase YAG:Eu^3+^ microspheres formed and were collected in a quartz boat; the off-gas was passed through a gas-washing bottle. The whole process is continuous and requires no post-annealing.

### 2.3. Characterisation

Crystal structure was characterised by X-ray diffraction (XRD, Thermo ARL XTRA, Thermo Fisher Scientific, Ecublens, Switzerland, Cu Kα, 10–80°). Morphology and surface elemental distribution were examined by field-emission scanning electron microscopy (FE-SEM, Zeiss ULTRA-55, Carl Zeiss AG, Oberkochen, Germany) with energy-dispersive X-ray (EDX) mapping. Nitrogen adsorption–desorption isotherms (Micromeritics ASAP 2020HD88, Micromeritics Instrument Corp., Norcross, GA, USA) provided the BET specific surface area and BJH pore-size distribution. Photoluminescence excitation (PLE) and emission (PL) spectra were recorded on a Hitachi F-4600 fluorescence spectrophotometer (Hitachi High-Technologies Corp., Tokyo, Japan). For a fair comparison of the three morphologies, equal volumes of powder were packed to the same level into an identical sample holder and measured under identical instrument settings (the same excitation wavelength, slit widths, photomultiplier voltage and scan parameters), so that the steady-state emission intensities of the solid, porous and hollow samples could be compared semi-quantitatively. Because equal powder volumes of solid and hollow particles do not necessarily contain the same mass or number of particles, and because the PL intensity of powders is also affected by packing density, scattering, self-absorption and excitation penetration depth [17], these intensities are not normalised per unit mass or per Eu content, and the comparison of the morphologies relies primarily on the absolute quantum yield and the ^5^D_0_ lifetime, which are much less sensitive to these factors. The absolute photoluminescence quantum yield under 250 nm excitation was determined with an integrating sphere for one representative sample of each morphology (S5, P3 and H3).

## 3. Results and Discussion

### 3.1. Low-Temperature Formation of Single-Phase YAG

**Figure 1:**
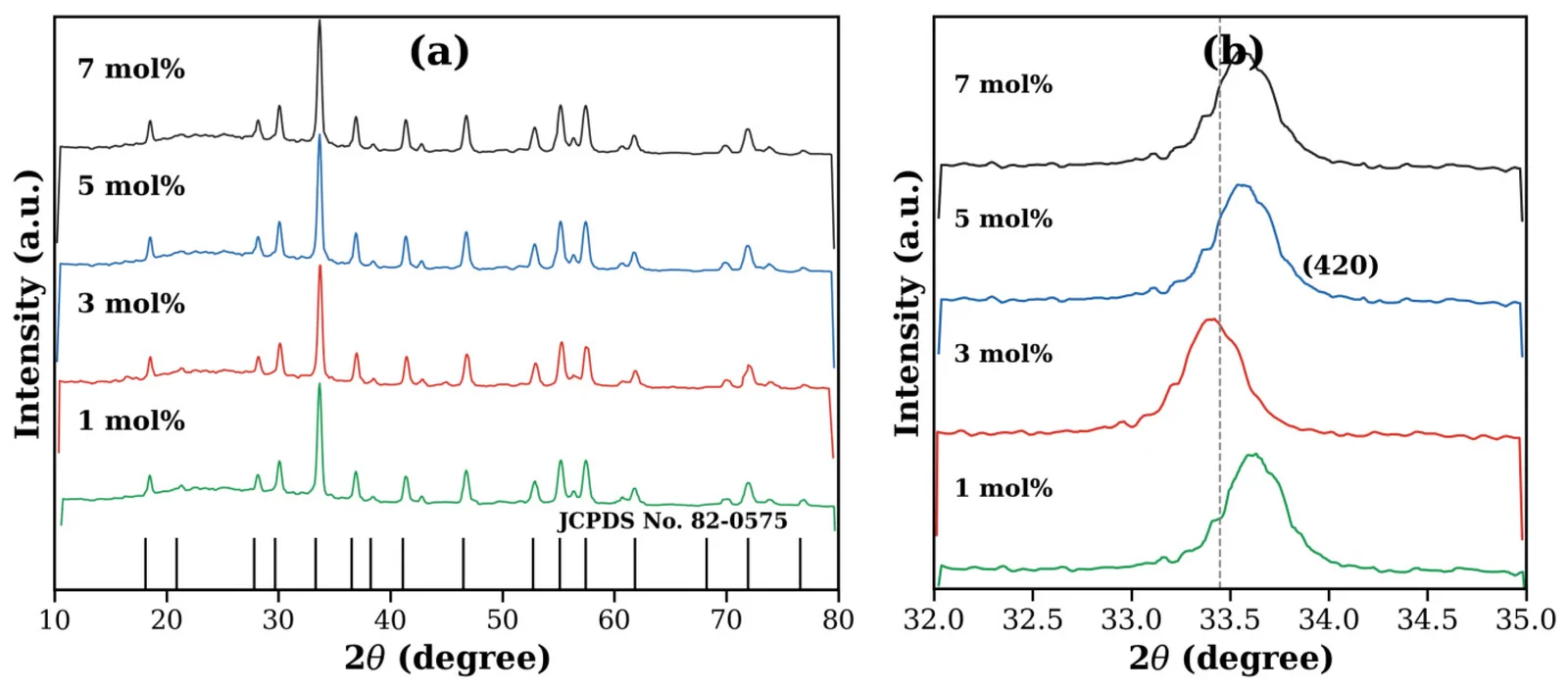
XRD patterns of YAG:Eu^3+^ microspheres with different Eu^3+^ contents: (**a**) full patterns; (**b**) magnified (420) reflection.

**Figure 2:**
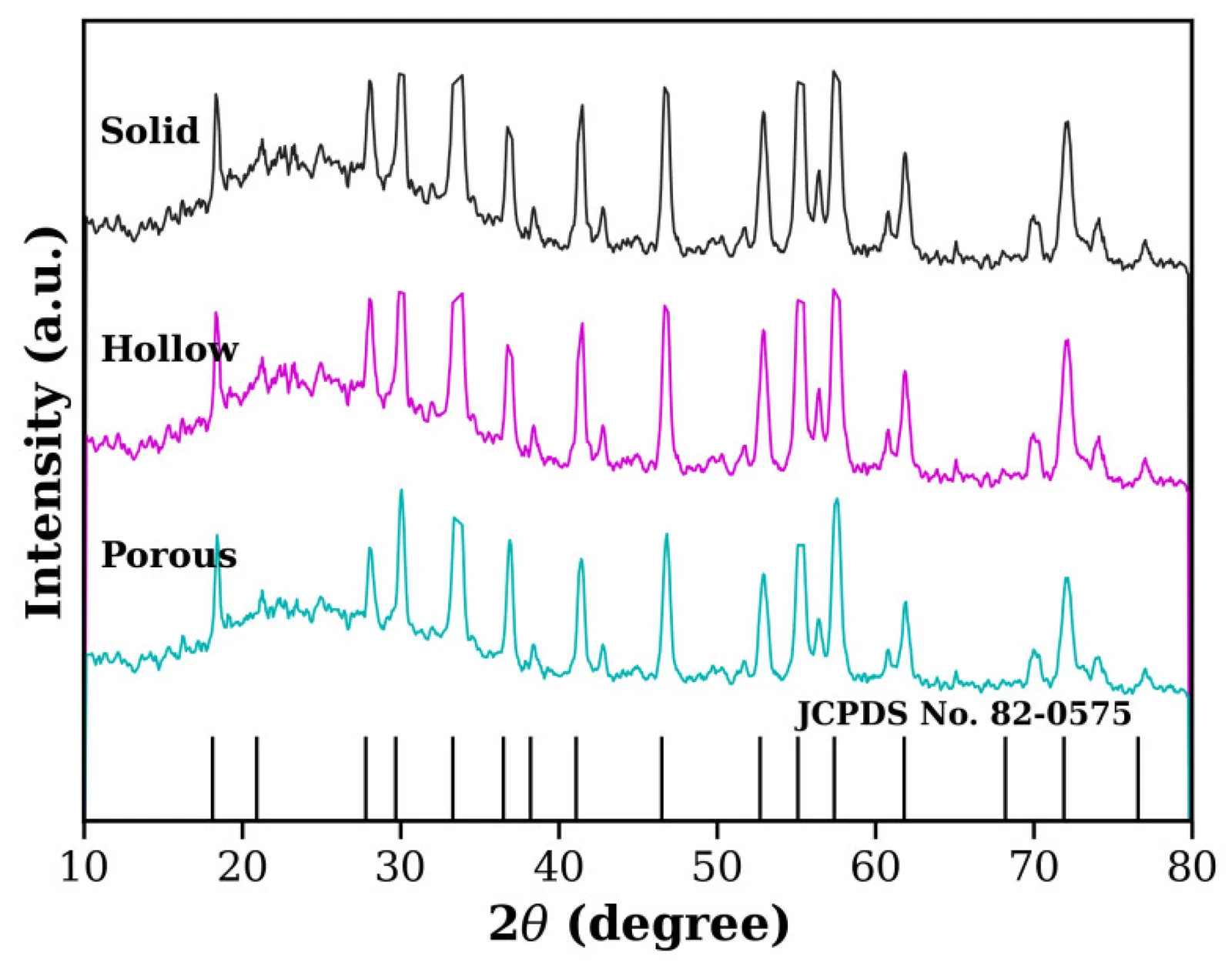
XRD patterns of solid, hollow and porous YAG:Eu^3+^ microspheres.

A central requirement of this work is that phase-pure YAG forms despite the low processing temperature. Figure 1a shows the XRD patterns of the additive-free YAG:Eu^3+^ microspheres with 1–7 mol% Eu^3+^. All patterns are sharp and match cubic YAG (JCPDS No. 82-0575) with no detectable impurity or intermediate (YAP/YAM) phases, confirming that YAG, single-phase within the detection limit of XRD, crystallised at only 900 °C—far below the >1600 °C of conventional solid-state synthesis. This can be attributed to each atomised droplet acting as a confined micro-reactor in which the cations are pre-mixed at a fixed stoichiometry over very short diffusion distances, so the solid-state reaction proceeds to completion at low temperature [15]. The weaker reflections at approximately 42.6°, 56.2° and 60.7° also belong to YAG, corresponding to the (440), (552)/(633)/(721) and (651) planes, which are allowed for the Ia-3d garnet structure but were omitted from the reference sticks because of their low intensity. The magnified (420) reflection (Figure 1b) shows only a small, non-monotonic variation with Eu^3+^ content. Although substitution of the larger Eu^3+^ (106.6 pm, CN = 8) for Y^3+^ (101.9 pm, CN = 8) is expected to expand the lattice [18], the resulting shift at these doping levels is within the uncertainty of single-peak measurements without an internal standard, so no systematic lattice expansion can be resolved. Importantly, the patterns of the solid, porous and hollow samples are essentially identical (Figure 2): neither citric acid nor sucrose alters the phase or crystallinity, so morphology can be engineered independently of the host crystal chemistry. The broad background at approximately 15–30° in Figure 2 is common to all three samples and is not accompanied by any additional reflection; a minor amorphous contribution below the XRD detection limit cannot, however, be completely excluded.

### 3.2. Morphology Control: Solid, Porous and Hollow Microspheres

**Figure 3:**
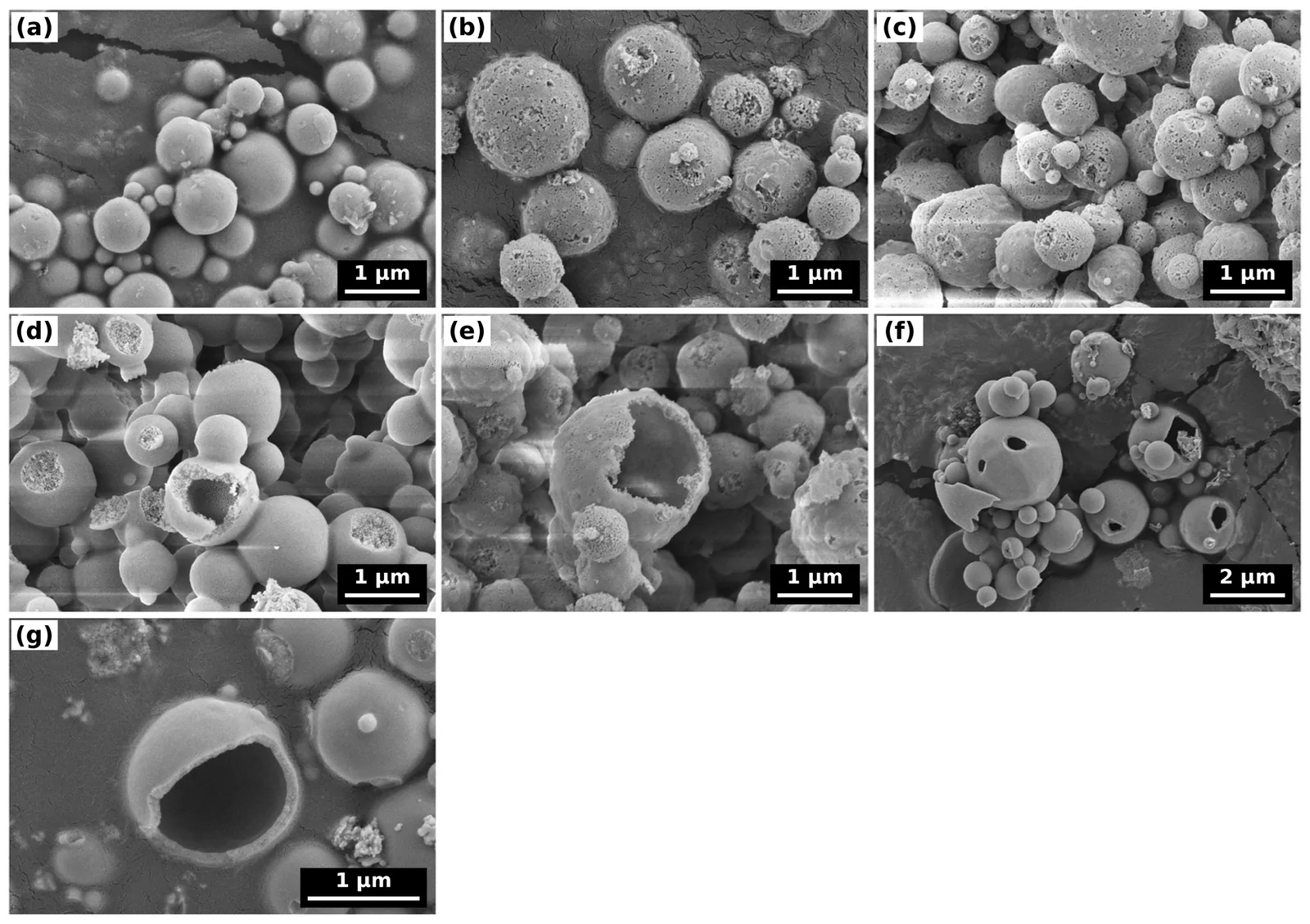
FE-SEM images of YAG:Eu^3+^ microspheres: (**a**) solid S5; (**b**–**d**) porous P1, P3, P5; (**e**–**g**) hollow H1, H3, H5. Fractured spheres in (**e**–**g**) reveal the hollow interior and thin shell, confirming the hollow architecture.

The defining feature of this route is that a single process yields three distinct morphologies through the choice of additive. FE-SEM images (Figure 3) show that all products are well-dispersed spheres in the sub-micrometre to micrometre range. The additive-free product (S5, Figure 3a) is solid with a smooth, intact surface. With citric acid (P1–P5, Figure 3b–d) the surfaces become increasingly porous as the additive concentration rises: the carboxyl groups are expected to chelate the metal ions and form a complex that is uniformly distributed in the drying droplet, and its thermal decomposition leaves behind a network of fine pores, so more citric acid produces larger pores. With sucrose (H1–H5, Figure 3e–g) the particles become hollow, and the SEM images indicate a tendency toward thinner shells with increasing sucrose content: sucrose is expected to form a film at the droplet surface that alters the evaporation path and drives the solute outward, generating a central cavity whose size grows—and whose wall thins—with sucrose content [16]. The two additives thus provide concentration-based control over the porosity and, as indicated qualitatively by SEM, the shell thickness.

**Figure 4:**
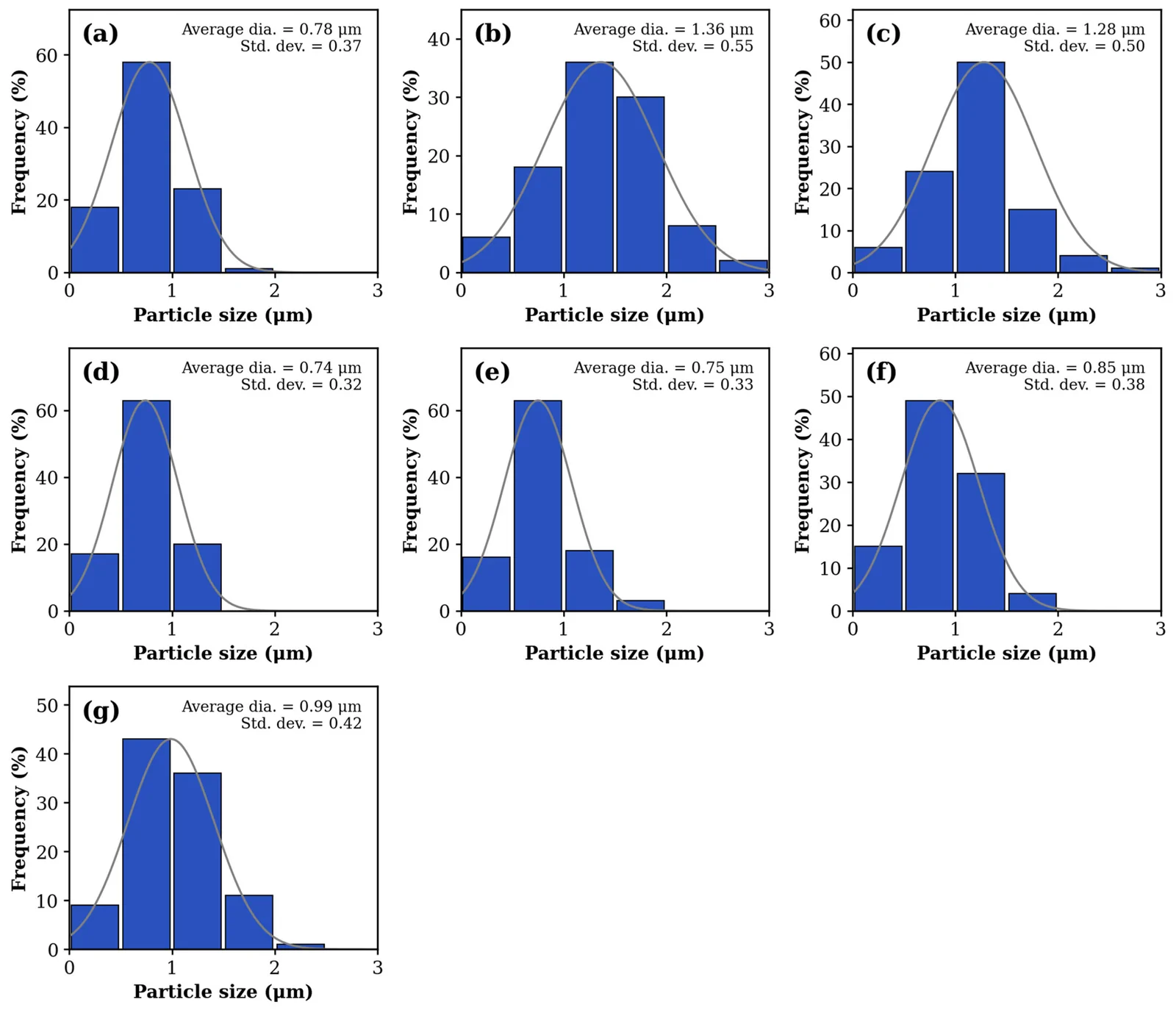
Particle-size distributions of YAG:Eu^3+^ microspheres: (**a**) solid S5; (**b**–**d**) porous P1, P3, P5; (**e**–**g**) hollow H1, H3, H5 (*n* = 100 particles per sample; mean ± SD given in each panel).

Statistical sizing of 100 particles per sample (Figure 4) gives unimodal distributions with mean diameters (±SD) of 0.78 ± 0.37, 1.36 ± 0.55, 1.28 ± 0.50, 0.74 ± 0.32, 0.75 ± 0.33, 0.85 ± 0.38 and 0.99 ± 0.42 μm for S5, P1, P3, P5, H1, H3 and H5, respectively; the Gaussian curves in Figure 4 are shown as a guide to the eye. All mean diameters lie within the 0.5–3.0 μm range generally regarded as suitable for phosphors. The comparable widths arise because, at fixed atomiser settings and precursor concentration, the droplet size is essentially constant [19]. Morphology engineering therefore does not come at the cost of size uniformity.

### 3.3. Role of the Organic Additives

**Figure 5:**
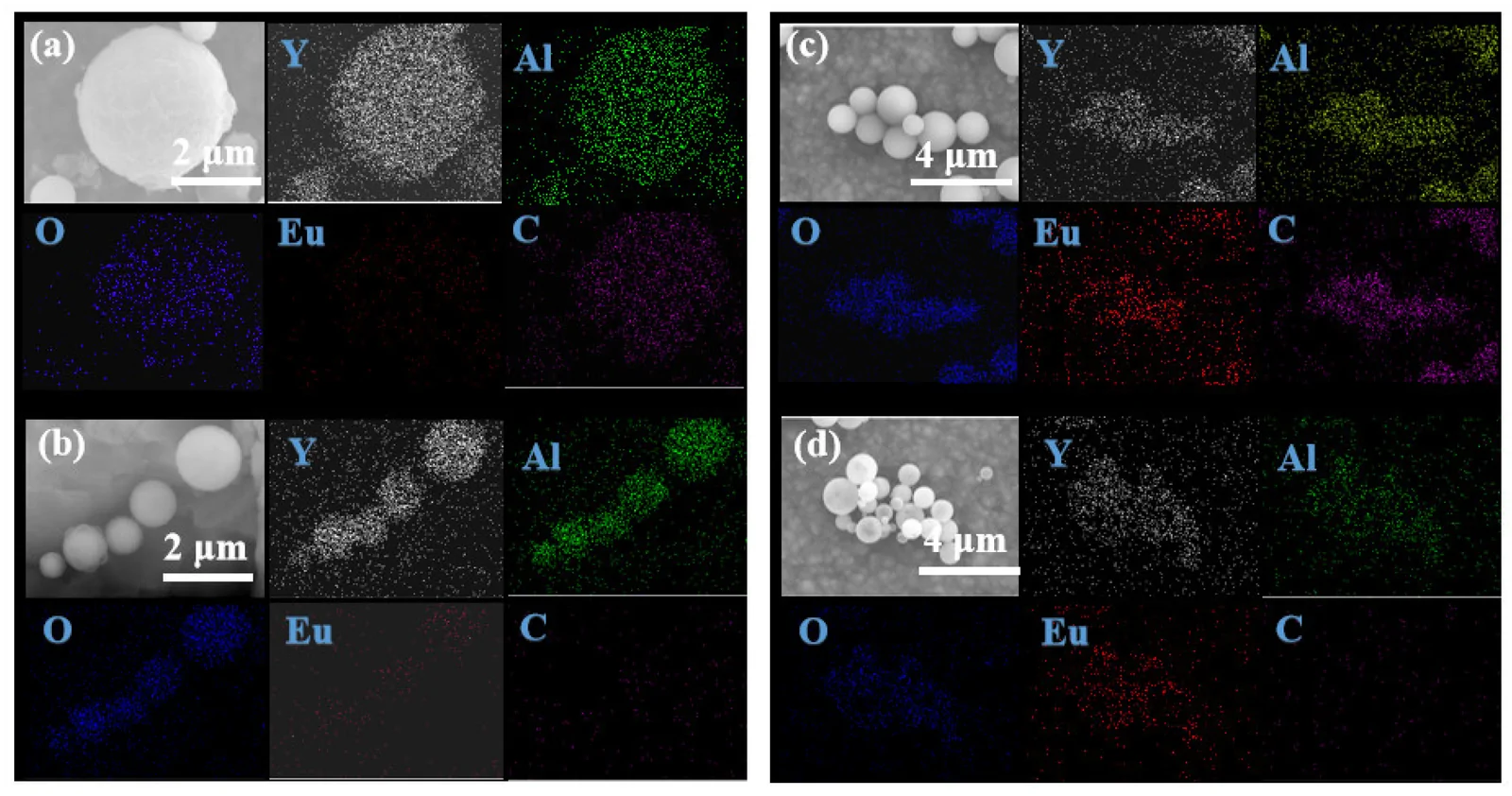
EDX elemental mapping of YAG:Eu^3+^ microspheres: (**a**) porous, 150 °C; (**b**) porous, 900 °C; (**c**) hollow, 150 °C; (**d**) hollow, 900 °C.

To verify that the additives are fully removed and to map the elemental distribution, the citric acid (P3) and sucrose (H3) precursors were sprayed at 150 °C and at 900 °C and analysed by EDX mapping (Figure 5). In every case Y, Al, O and Eu are uniformly distributed and coincide with the particle outline, indicating a homogeneous distribution of the activator. At 150 °C a strong, uniformly distributed carbon signal is present, showing that the organic additive is retained at this stage, consistent with its templating role; at 900 °C the carbon signal nearly vanishes, indicating that the additive decomposes almost completely during synthesis and leaves the porous (citric acid) or hollow (sucrose) architecture behind. This is consistent with the proposed pore- and film-forming mechanisms, and no carbon signal is detectable by EDX in the final phosphor.

**Figure 6:**
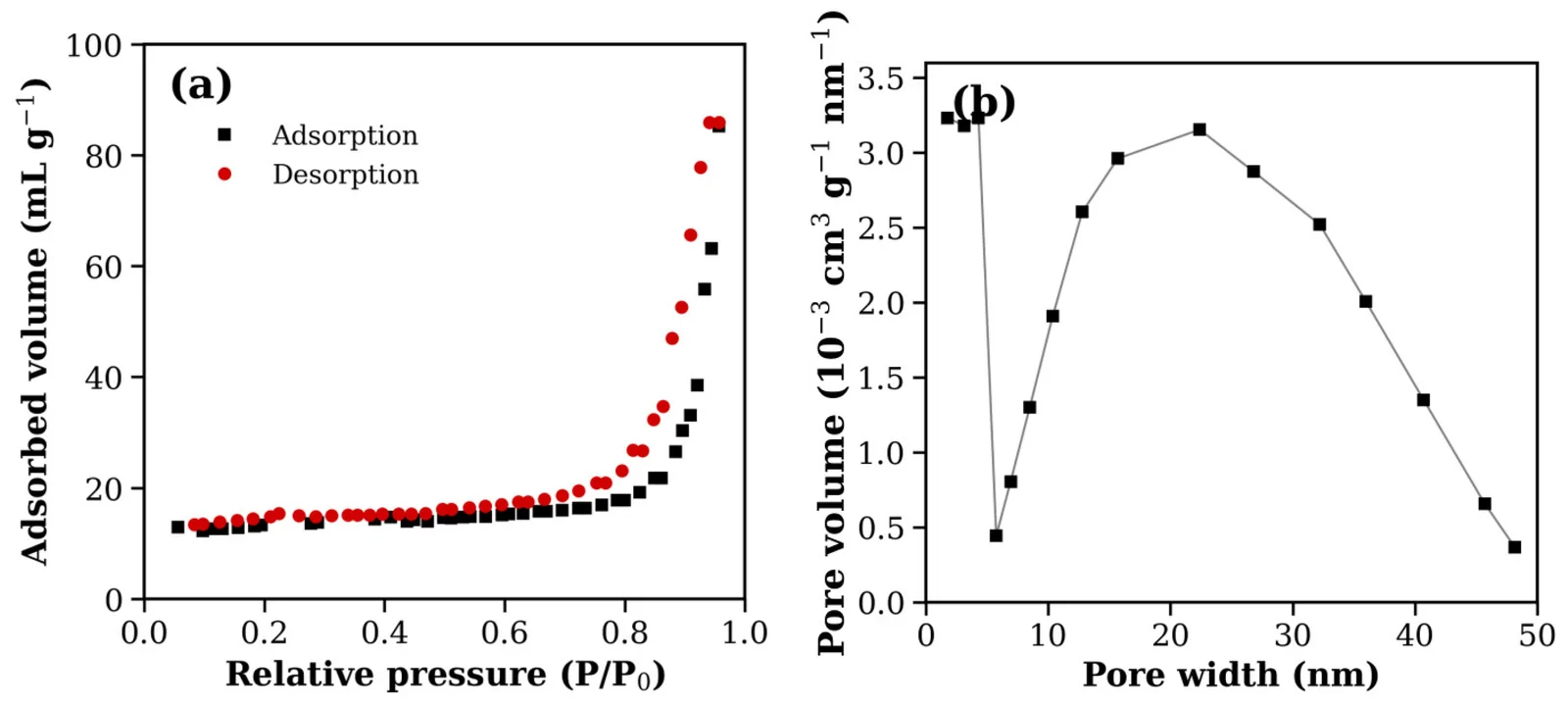
Porous YAG:Eu^3+^ microspheres: (**a**) N_2_ adsorption–desorption isotherm; (**b**) pore-size distribution.

Nitrogen physisorption quantifies the porous texture (Figure 6). The isotherm is type IV(a) with an H3 hysteresis loop characteristic of slit-shaped mesopores formed by aggregated plate-like domains [20], and the BJH distribution spans 6–46 nm. The porous sample has a BET specific surface area of 23.65 m^2^ g^−1^ and an average pore width of 28.52 nm, confirming an abundant, accessible mesopore network that is consistent with the FE-SEM observations. Since the BJH pore-size distribution depends on the assumptions of the underlying model (cylindrical pores and the Kelvin equation), these pore sizes should be regarded as model-dependent estimates [20].

The fractured hollow spheres in Figure 3g reveal a smooth, compact shell wall with no visible intrawall porosity, indicating that the sucrose-derived shell is dense rather than mesoporous. This is consistent with the sucrose film-formation mechanism described in Section 3.6, in which sucrose concentrates at the droplet surface and solidifies into a compact crust. The dense, low-defect character of the shell is further supported by the luminescence lifetime data (Section 3.4): the ^5^D_0_ lifetime of the hollow H3 sample (τ = 1.76 ms) is within 3% of that of the solid S5 (τ = 1.82 ms), indicating that the hollow architecture does not introduce significant non-radiative quenching. Consequently, the ~4% reduction in emission intensity of H3 relative to S5 is unlikely to arise from surface-defect quenching and is consistent with the removed core being largely optically inactive—so that hollowing barely lowers the emission—as elaborated in Section 3.5.

### 3.4. Luminescence and Activator Optimisation

**Figure 7:**
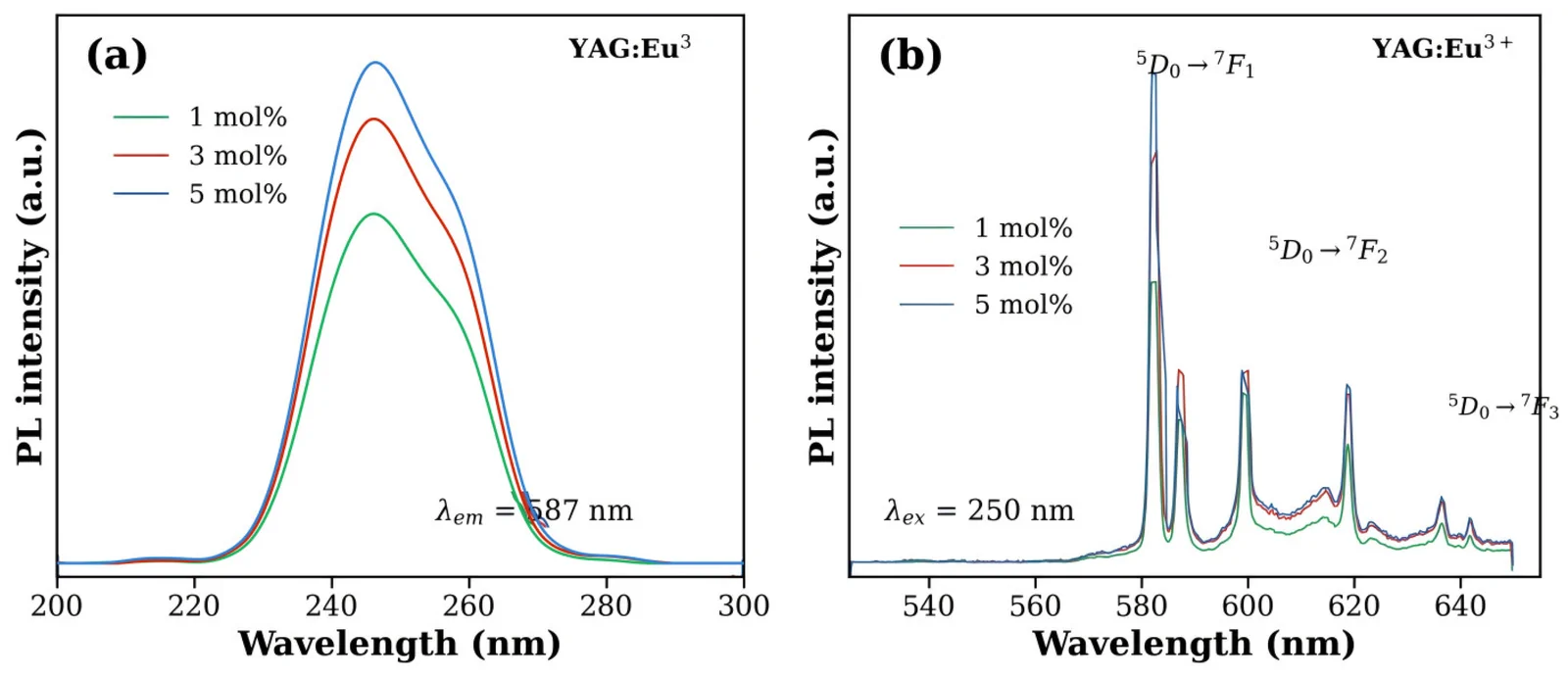
Photoluminescence spectra of YAG:Eu^3+^ with different Eu^3+^ contents: (**a**) excitation; (**b**) emission.

**Figure 8:**
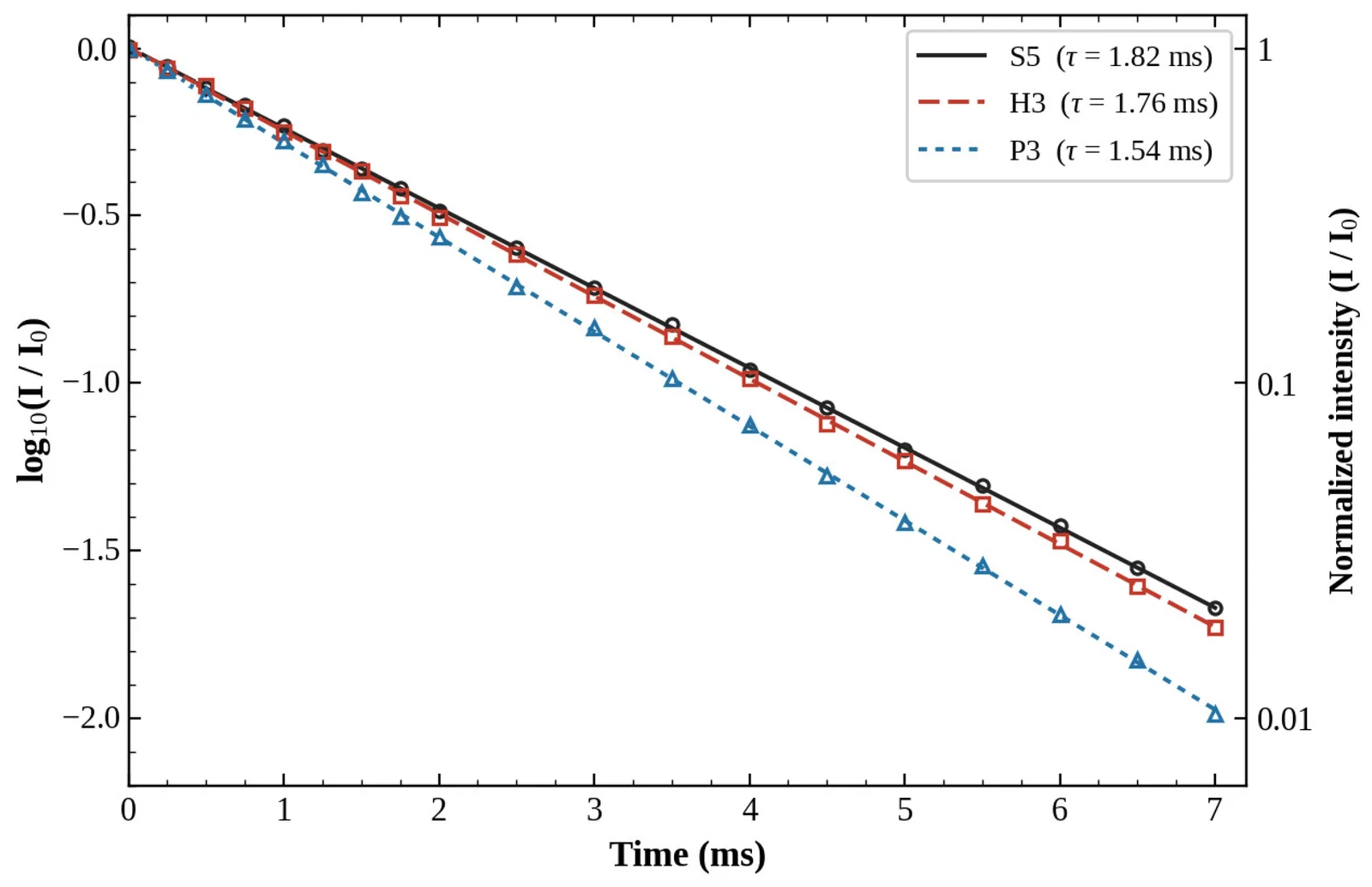
Semi-logarithmic ^5^D_0_ decay curves (λ^em^ = 587 nm, λ^ex^ = 250 nm) of solid S5, hollow H3 and porous P3 microspheres; open symbols are the measured data (circles, S5; squares, H3; triangles, P3) and the lines (solid, dashed and dotted, respectively) are single-exponential fits with the fitted lifetimes τ.

**Figure 9:**
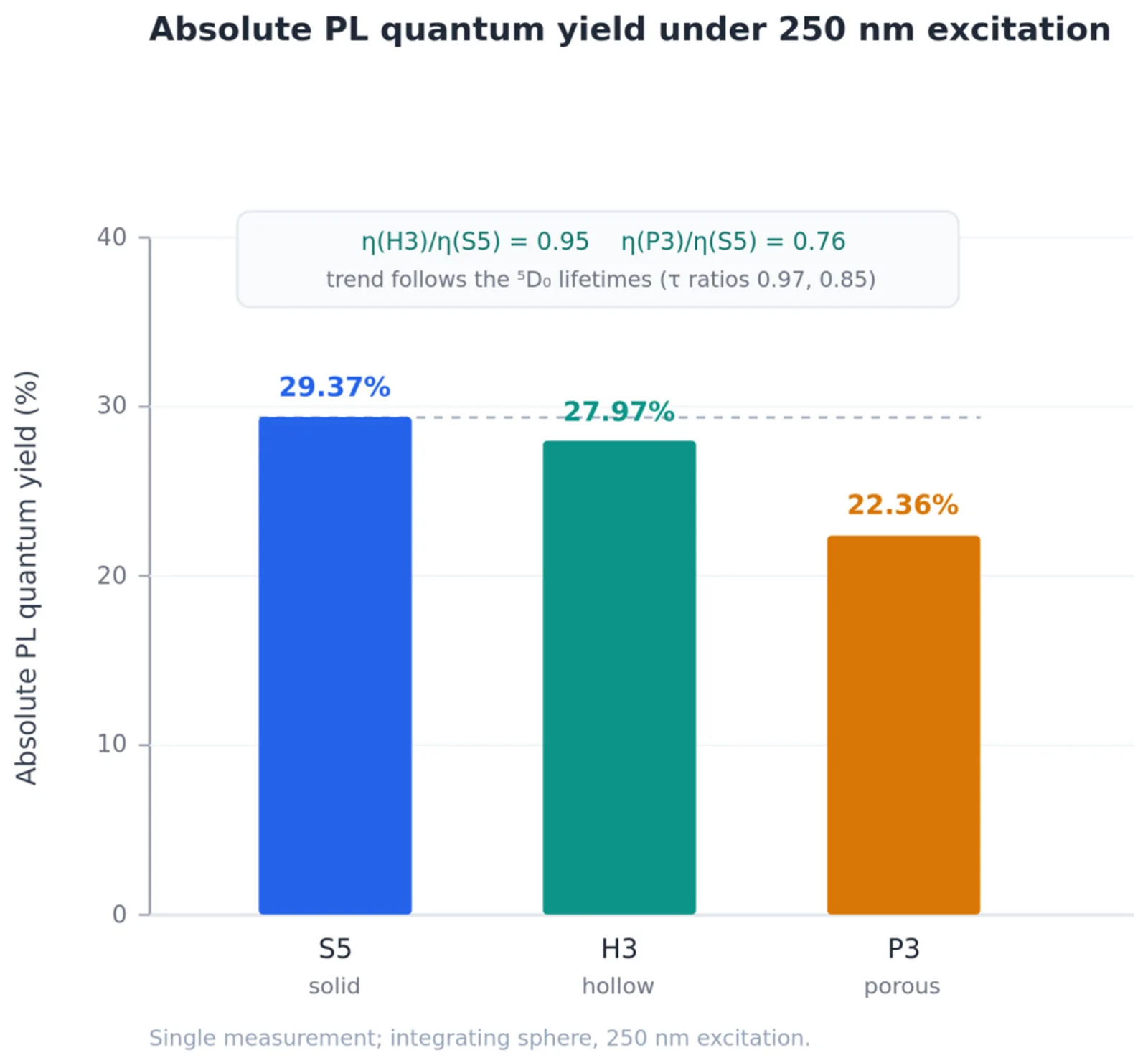
Absolute photoluminescence quantum yield (η, 250 nm excitation) of solid S5, hollow H3 and porous P3 YAG:Eu^3+^ microspheres. Values are from a single integrating-sphere measurement; the dashed line marks the solid-sample level. The hollow sample retains 95.2% of the solid-sample quantum yield, whereas the porous sample falls to 76.1%.

Figure 7 presents the photoluminescence of the additive-free series. The PLE spectra (Figure 7a) show a broad band at 220–260 nm peaking at 250 nm, assigned to the O^2−^–Eu^3+^ charge-transfer band. The excitation spectra were recorded in the charge-transfer region (200–300 nm) used for all emission measurements; the weaker intra-4f^6^ lines at longer wavelengths (e.g., ~393 and ~465 nm) were not covered. Under 250 nm excitation the emission (Figure 7b) consists of the characteristic Eu^3+^ lines in the 550–700 nm region: the magnetic-dipole ^5^D_0_→^7^F_1_ transition (587, 592 nm), the forced electric-dipole ^5^D_0_→^7^F_2_ transition (605, 626 nm) and the weaker ^5^D_0_→^7^F_3_ transition (645, 651 nm), reflecting Eu^3+^ at the D_2_ Y^3+^ site of the garnet lattice [21]. The emission intensity rises with Eu^3+^ content up to 5 mol% and then falls owing to concentration quenching; 5 mol% is therefore the optimum activator level and was used for all morphology-controlled samples. In YAG:Eu^3+^ the ^5^D_0_ lifetime shortens with increasing Eu^3+^ concentration owing to enhanced non-radiative energy migration to quenching sites [22]; since S5, P3 and H3 contain the same 5 mol% Eu^3+^, this concentration-related contribution is common to all three and does not affect the comparison of the morphologies based on their lifetimes and quantum yields (Figure 8 and Figure 9).

To verify that the distinct morphologies do not perturb the Eu^3+^ crystal-field environment, the ^5^D_0_ luminescence decay curves of S5, P3 and H3 were measured at λ^em^ = 587 nm under pulsed 250 nm excitation (Figure 8). Over the fitted range (0–7 ms), the decays of all three samples are well described by a single-exponential function (R^2^ > 0.999), consistent with Eu^3+^ occupying a single dominant D_2_ dodecahedral site in the YAG garnet lattice. The fitted values are used as effective lifetimes for comparing the samples rather than as proof of a strictly single-site decay. The fitted lifetimes are τ(S5) = 1.82 ms, τ(H3) = 1.76 ms and τ(P3) = 1.54 ms. The lifetime of the hollow H3 sample is within 3% of that of the solid S5, indicating that the hollowing process does not introduce significant non-radiative quenching pathways or alter the local Eu^3+^ site symmetry. The shorter lifetime of the porous P3 sample (τ = 1.54 ms, 84.6% of S5) is attributed to non-radiative relaxation at the abundant mesopore surface defects (BET area 23.65 m^2^ g^−1^, Section 3.3), consistent with its lower steady-state emission intensity in Table 2. Together, the lifetime data indicate that the ~4% intensity reduction observed for H3 relative to S5 reflects the marginally lower quantum yield rather than any quenching or structural disorder introduced by hollowing—supporting the shell-emission picture of Section 3.5, in which the near-equal emission arises because the removed core is largely optically inactive.

To examine directly, and not only through the decay kinetics, that the hollow architecture preserves the intrinsic luminescence efficiency, the absolute photoluminescence quantum yields (η) of the solid (S5), hollow (H3) and porous (P3) microspheres were measured under 250 nm excitation in an integrating sphere (Figure 9). The solid and hollow samples show similar quantum yields, η(S5) = 29.37% and η(H3) = 27.97%, the hollow value being 95.2% of the solid one, whereas the porous sample is markedly lower, η(P3) = 22.36% (76.1% of S5). Because each value was obtained from a single integrating-sphere measurement, the 4.8% difference between S5 and H3 is not interpreted further; the relevant observation is that H3 retains 95% of the quantum yield of S5, whereas P3 retains only 76%. This ordering follows the same trend as the ^5^D_0_ lifetimes (τ(S5) = 1.82 ms, τ(H3) = 1.76 ms, τ(P3) = 1.54 ms; ratios 96.7% and 84.6%): the near-equal quantum yields of the solid and hollow samples indicate that hollowing introduces no major additional non-radiative decay, in agreement with the lifetime data. For the porous sample the relative reduction in η is somewhat larger than that in τ, consistent with extra non-radiative loss at its extended mesopore surface (BET specific surface area 23.65 m^2^ g^−1^, Section 3.3), which depresses the steady-state efficiency beyond what the lifetime alone reflects. Crucially, because η(H3) ≈ η(S5), the ~4% lower steady-state emission of H3 relative to S5 (Table 2) is consistent with their slightly different quantum yields rather than a material shortfall; indeed, because the hollow sample contains only ~57% of the rare-earth material yet emits almost as strongly (96.1%, Table 2), the ~43% of material removed as the core appears to contribute little to the emission of the solid particle. This is precisely the premise underlying the spherical-shell rare-earth-saving model developed in Section 3.5, and it identifies the hollow—rather than the porous—architecture as the efficient route to rare-earth saving.

### 3.5. Rare-Earth Saving by the Hollow Architecture

**Figure 10:**
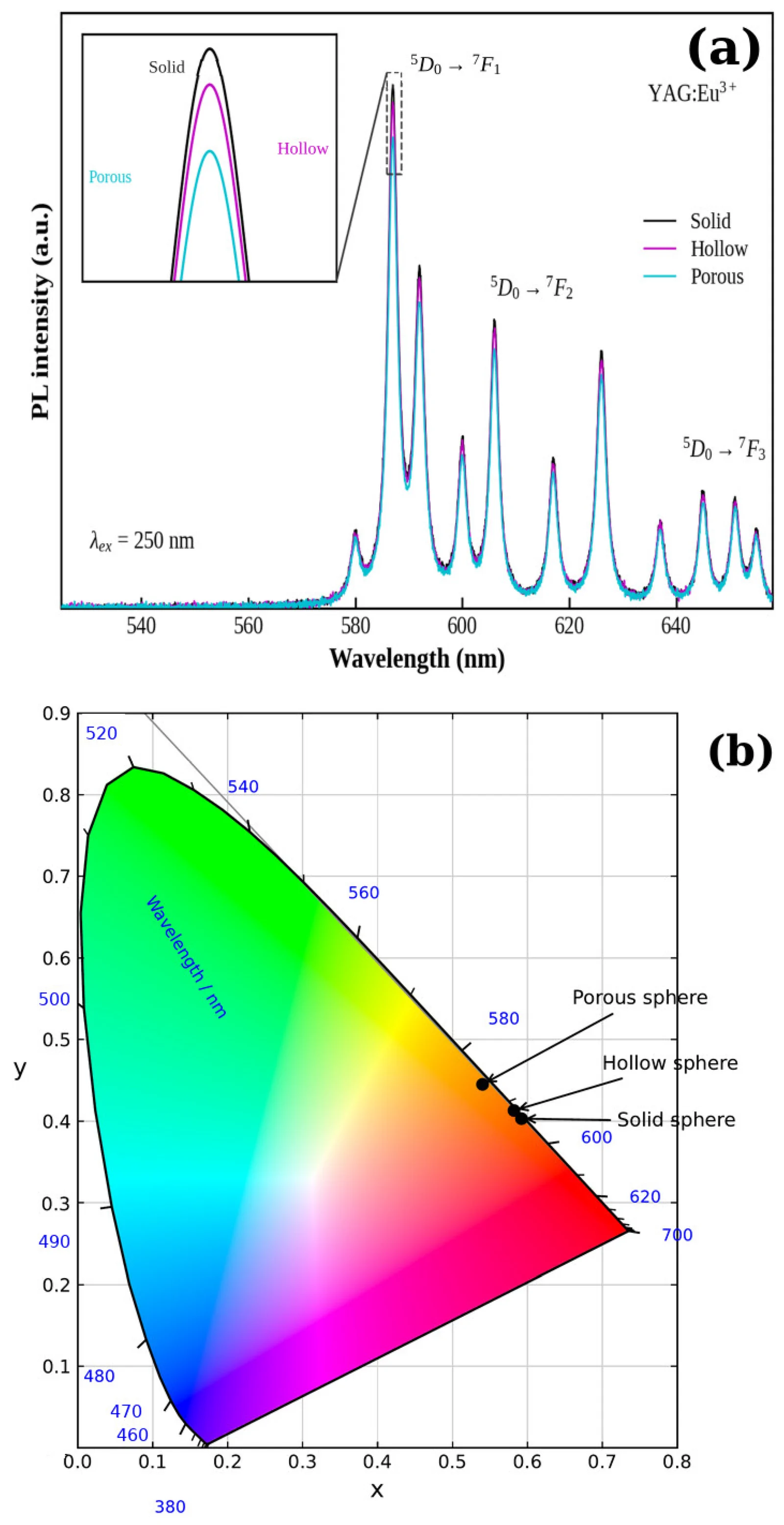
Photoluminescence of YAG:Eu^3+^ with different morphologies: (**a**) emission; (**b**) CIE chromaticity diagram.

The central result is summarised in Figure 10 and Table 2. The emission peak positions of the solid (S5), porous (P3) and hollow (H3) samples coincide (Figure 10a), indicating that the morphology does not significantly perturb the Eu^3+^ coordination environment. This is supported by the asymmetry ratio AR = I(^5^D_0_→^7^F_2_)/I(^5^D_0_→^7^F_1_): estimated from the integrated intensities of the spectra in Figure 10a, AR is approximately 0.8 for all three samples (0.75–0.85, a spread within the uncertainty of the estimate). The magnetic-dipole ^5^D_0_→^7^F_1_ transition thus dominates, as expected for Eu^3+^ on the D_2_ dodecahedral site of the garnet lattice, and AR is close to the value of 0.75 reported for YAG:Eu^3+^ [23]. The absence of a significant difference among the solid, porous and hollow samples indicates that neither the additives nor the morphology introduce detectable Eu^3+^ segregation or a distinct low-symmetry (e.g., amorphous-phase) Eu^3+^ environment. Because the three samples carry the same Eu^3+^ molar fraction, the observed intensity is governed by both the amount of emitting material and the radiative efficiency. Because the solid and hollow samples have essentially the same efficiency (Figure 8 and Figure 9), the hollow sample emits almost as strongly as the solid one (96.1%, Table 2) despite containing only ~57% of the material, which indicates that the material removed as the central core contributes little to the emission; the porous sample, although it contains more material than the thin hollow shell, emits less than the hollow one owing to the extra non-radiative loss at its mesopore surfaces (its lower quantum yield and lifetime). The 587 nm intensity therefore decreases in the order solid > hollow > porous (Table 2) [24]. Because the lifetime and quantum-yield measurements (Figure 8 and Figure 9) indicate that the Eu^3+^ crystal-field environment and luminescence efficiency are essentially unchanged between the solid and hollow samples, their near-equal emission—despite the hollow sample using ~43% less material—indicates that the removed core is largely optically inactive, so the rare-earth saving can be quantified geometrically with the spherical-shell volume model developed below. Crucially, the hollow microspheres retain almost the full emission output: as listed in Table 2, the strongest 587 nm line of the hollow microspheres reaches 96.1% of that of the solid microspheres. The CIE coordinates of all three samples lie in the orange–red region (Figure 10b), with the denser hollow and solid spheres showing slightly higher colour purity than the loosely packed porous spheres [9].

This near-equivalence in brightness, combined with the removal of the optically inactive core, is what makes the hollow architecture attractive for rare-earth saving. The amount of raw material saved can be estimated from a spherical-shell model. Because the Y/Al/Eu ratio is fixed, the fractional saving applies equally to the rare-earth (Y and Eu) precursors and to the YAG:Eu^3+^ material as a whole. Treating a hollow microsphere as a uniform shell of outer diameter *D* and wall thickness *t*, and assuming the same composition and density as a solid sphere of equal outer diameter, the material saving equals the volume fraction of the central cavity:

*R* = 1 − *V*_shell_/*V*_solid_ = (1 − 2*t*/*D*)^3^ (1)

Using the wall thickness of the H3 hollow sample measured directly from the SEM images of fractured spheres (*t* ≈ 105 nm) and the experimental outer-diameter range of 0.5–3.0 μm (Figure 4), Equation (1) predicts a saving that grows with particle size, from about 43% at the mean diameter of the H3 powder (*D* ≈ 0.85 μm) to 77–80% for the largest thin-walled spheres (*D* ≈ 2.5–3.0 μm). In other words, the hollow YAG:Eu^3+^ microspheres preserve 96.1% of the emission while, for the H3 sample on which this emission was measured (mean outer diameter ≈ 0.85 μm and shell thickness *t* ≈ 105 nm from fractured-sphere SEM), saving ~43% of the rare-earth-bearing raw material according to Equation (1), the saving increases monotonically with outer diameter at fixed shell thickness, reaching ~77–80% in the thin-shell limit (2.5–3.0 μm). Viewed from a material-efficiency perspective, the trade-off is favourable even on this same-population basis: delivering 96.1% of the emission with ~57% of the material corresponds to a geometrically estimated emission-per-material gain of approximately 1.68 relative to solid spheres, and the advantage grows further for larger, thinner-shelled particles, beyond the sub-micron sizes realised here. This quantitative advantage, achieved in a single continuous step at 900 °C, is the principal merit of the present route over both solid phosphors and the multi-step template/hydrothermal hollow-phosphor routes reported previously [9,10,11].

Equation (1) is a first-order geometric approximation that assumes (i) spherical particles with a uniform shell thickness, (ii) a shell with the same composition and density as the solid particles, and (iii) no significant compositional gradients or structural defects in the shell. Assumption (ii) is supported by the uniform Eu distribution in the EDX maps of the hollow sample (Figure 5c,d) and by the similar lifetimes and quantum yields of S5 and H3 (Figure 8 and Figure 9); a lower shell density or residual porosity would increase the actual saving, whereas a non-uniform shell thickness may shift the estimate in either direction. The estimate is most sensitive to the shell thickness: for *D* = 0.85 μm, an assumed uncertainty of ±20 nm in *t* changes the calculated saving from ~51% (*t* = 85 nm) to ~35% (*t* = 125 nm). With respect to the particle-size distribution, evaluating Equation (1) at the number-mean diameter is conservative: because the material content of a particle scales with *D*^3^, weighting Equation (1) over the measured size distribution of H3 (Figure 4f, *t* = 105 nm) gives a volume-weighted cavity fraction of approximately 56%. The ~43% saving should therefore be regarded as a geometric estimate, whereas the ~77–80% values for 2.5–3.0 μm spheres are a projection beyond the size range of the present H3 powder.

A natural question is why the hollow microspheres, which contain only ~57% of the rare-earth material of the solid spheres, nonetheless retain 96.1% of their emission. This near-equivalence implies that the removed central core contributes only marginally to the observed emission—that is, the emission originates predominantly in the outer region of the particle. Such behaviour is physically reasonable for Eu^3+^ phosphors excited within the O^2−^–Eu^3+^ charge-transfer band: the strong, broad absorption around 250 nm (Figure 7a) is attenuated over a short distance into the particle, so the excitation is deposited mainly in a near-surface layer of thickness comparable to the shell, leaving the core weakly excited. It is also consistent with previous reports that hollow, yolk–shell and thin-shell Eu^3+^ microspheres retain—or, through enhanced internal light scattering, even exceed—the emission of their fully dense counterparts [16,25,26]. We do not claim direct spatially resolved evidence for shell-confined emission in the present samples; rather, the concurrence of near-equal quantum yield and lifetime (Figure 8 and Figure 9) with near-equal steady-state intensity is most simply explained by emission that is concentrated in the outer shell. Under this picture the hollow architecture removes essentially optically inactive material, which is what makes the geometrically estimated ~1.68-fold emission-per-material gain possible.

### 3.6. Formation Mechanism

The combined XRD, FE-SEM, EDX and physisorption results are consistent with two well-documented mechanisms of morphology control in spray pyrolysis, as described below.

For the hollow microspheres (sucrose series), the key parameter is the Péclet number Pe = *r*^2^/*D*_s_*τ*, where *r* is the droplet radius, *D*_s_ is the solute diffusivity and *τ* is the evaporation time [15,27]. When Pe ≫ 1, solvent evaporates much faster than the solute can diffuse inward; the solute therefore accumulates at the droplet surface, eventually forming a rigid crust that locks in a central void. Sucrose promotes this regime because its large molecular weight greatly reduces *D*_s_, and its hydroxyl groups also stiffen the surface layer through hydrogen bonding, facilitating early crust formation [16]. The actual Pe values were not evaluated quantitatively in this work; the formation of hollow particles in the sucrose series is therefore described as consistent with a high-Pe regime rather than as a demonstration of it. As the sucrose concentration increases the surface film forms earlier and traps a larger cavity, so the shell thins progressively (Figure 3e–g), consistent with the EDX observation that carbon is uniformly distributed in the 150 °C product but no longer detectable at 900 °C (Figure 5c,d). Three factors are expected to contribute to the formation and stabilisation of the central cavity. First, surface enrichment: at high Pe the solute reaches its critical supersaturation at the droplet surface long before it does at the centre, so precipitation starts as a shell whose thickness is set by the concentration gradient at the onset of precipitation [15,27,28]. Second, gas evolution inside the shell: once the crust has formed, the remaining water evaporates and sucrose and the metal nitrates decompose (releasing H_2_O, CO_2_ and NO_x_) inside the particle. If the crust is permeable, these gases escape and the void is preserved; if it is less permeable, the internal gas pressure inflates the particle, enlarging the cavity and thinning the wall [28,29]. The openings seen in some of the fractured shells in Figure 3f are consistent with such gas release. Third, shell rigidity: the hydrogen-bonded, sucrose-rich crust and, at higher temperature, the carbonaceous residue formed by its partial decomposition (strong C signal at 150 °C, Figure 5c) stiffen the shell so that it does not collapse as drying continues, until the inorganic framework crystallises into YAG and the carbon is burnt off (Figure 5d). Without sucrose the crust forms late and remains too thin and weak to support a void, so the particle shrinks into a dense sphere.

For the porous microspheres (citric acid series), the mechanism is different. Kinoshita et al. demonstrated that when citric acid is added to a spray-pyrolysis precursor, it precipitates before the inorganic salts owing to its lower solubility, so it acts as a distributed template that occupies space uniformly throughout the droplet interior; on subsequent heating the citric acid oxidises to CO_2_ and H_2_O at temperatures below 500 °C—well before oxide sintering—leaving behind a network of fine pores whose size and density scale with the additive loading [30]. This is consistent with the progressive increase in pore size from P1 to P5 (Figure 3b–d) and with the measured BET surface area of 23.65 m^2^ g^−1^ and mean pore width of 28.52 nm (Figure 6). In addition, the chelation of metal ions by the carboxylate groups of citric acid is expected to promote a homogeneous cation distribution that favours low-temperature, uniform crystallisation of single-phase YAG [18], consistent with the sharp XRD patterns of all porous samples (Figure 2).

**Figure 11:**
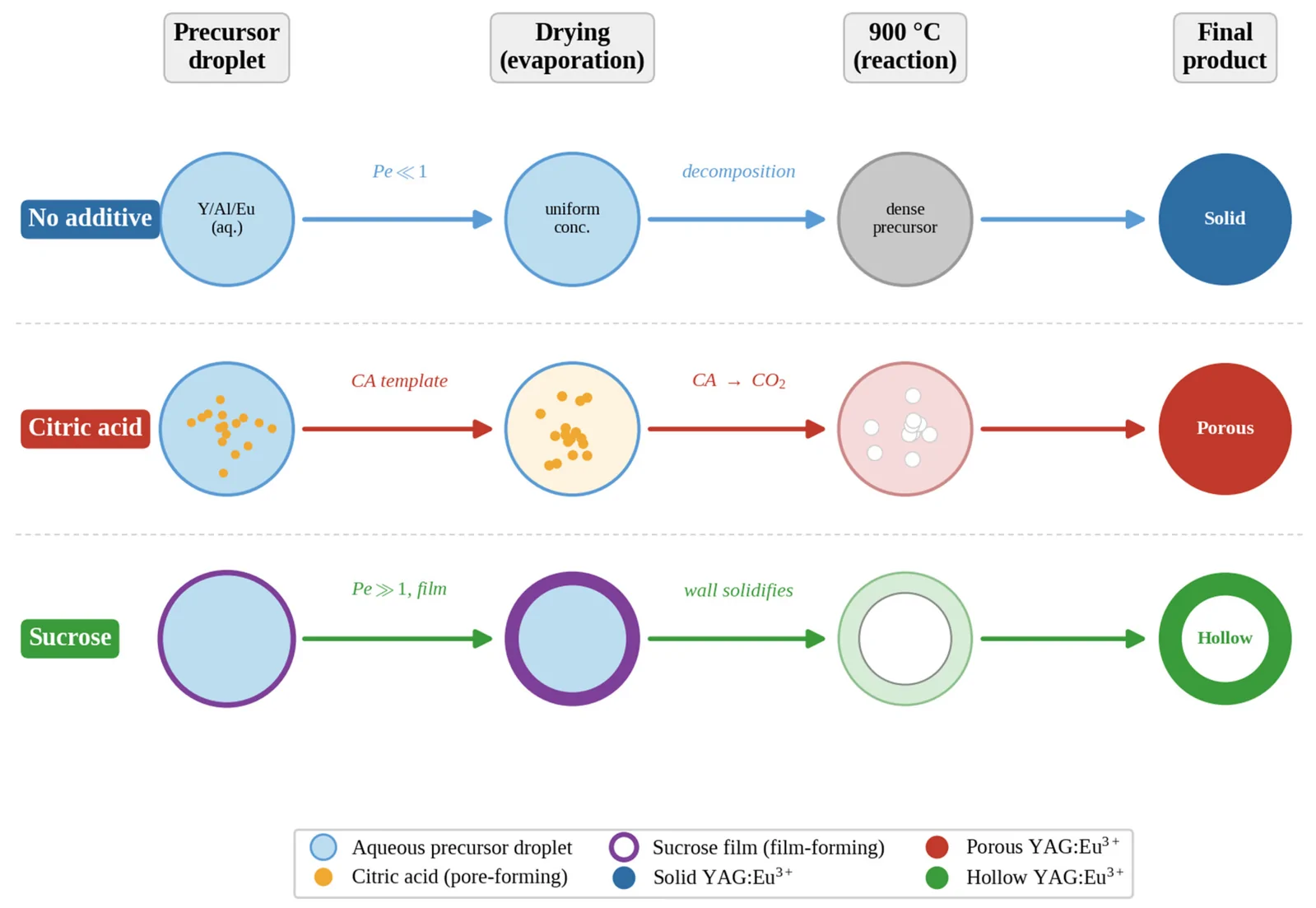
Schematic formation mechanisms of solid (no additive, Pe ≪ 1), porous (citric acid template) and hollow (sucrose film, Pe ≫ 1) YAG:Eu^3+^ microspheres by ultrasonic spray pyrolysis.

In the additive-free case (solid microspheres), the dense morphology is consistent with a lower Pe, for which the solute has time to diffuse inward during droplet shrinkage, so the concentration remains nearly uniform throughout and a dense solid particle results [15]. The three morphologies can thus be obtained by adding sucrose (hollow), citric acid (porous), or no additive (solid), giving the process its versatility. The proposed mechanisms are summarised schematically in Figure 11.

## 4. Conclusions

A one-step, continuous ultrasonic spray pyrolysis route has been developed that fabricates solid, porous and hollow YAG:Eu^3+^ microspheres from the same general process at only 900 °C, using citric acid as a pore-forming agent and sucrose as a film-forming agent. (i) Phase-pure cubic YAG formed for all morphologies without post-annealing, far below the conventional >1600 °C. (ii) The additive concentration controls the surface porosity (BET area 23.65 m^2^ g^−1^, mean pore width 28.52 nm) and, as indicated by SEM, the shell thickness, while the mean particle diameters remain within 0.74–1.36 μm. (iii) At the optimum 5 mol% Eu^3+^, on the same particle population the hollow microspheres retain 96.1% of the emission intensity of the solid ones while consuming, according to a spherical-shell geometric estimate, only ~57% of the rare-earth-bearing raw material (a ~43% saving)—equivalent to a geometrically estimated ~1.68-fold gain in emission per unit of rare-earth material, the hollow shell retaining essentially the same absolute quantum yield as the solid particle (27.97% versus 29.37%)—while a spherical-shell model (Equation (1)) projects a saving of up to ~77–80% for larger thin-walled spheres. (iv) The process is compatible with continuous operation and potentially scalable, unlike multi-step template and hydrothermal routes. The work thus provides a practical route to reducing the rare-earth raw material required for producing rare-earth red phosphors, and the additive-controlled USP approach should be extendable to other rare-earth phosphors and functional oxides.

## Figures and Tables

**Table 1:** Precursor compositions and the corresponding YAG:Eu^3+^ products.

Sample	Y(NO_3_)_3_/g	Al(NO_3_)_3_/g	Eu(NO_3_)_3_/g	H_2_O/mL	Sucrose/g	Citric Acid/g	Product
S1	7.52	12.40	0.07	180	–	–	Solid
S3	7.23	12.43	0.34	180	–	–	Solid
S5	7.04	12.48	0.47	180	–	–	Solid
S7	6.87	12.46	0.67	180	–	–	Solid
P1	7.04	12.48	0.47	179	–	1	Porous
P3	7.04	12.48	0.47	177	–	3	Porous
P5	7.04	12.48	0.47	175	–	5	Porous
H1	7.04	12.48	0.47	179	1	–	Hollow
H3	7.04	12.48	0.47	177	3	–	Hollow
H5	7.04	12.48	0.47	175	5	–	Hollow

**Table 2:** Strongest emission intensity (587 nm) of YAG:Eu^3+^ microspheres under 250 nm excitation. Intensities are given in arbitrary units (a.u.) as recorded by the spectrophotometer under identical acquisition settings (Section 2.3).

Solid YAG:Eu^3+^	Porous YAG:Eu^3+^	Hollow YAG:Eu^3+^
11,500,669	8,849,320	11,052,154

## Data Availability

The original contributions presented in this study are included in the article. Further inquiries can be directed to the corresponding authors.
